# Clinical Presentation and Course of Pulmonary Involvement in Chronic Nonbacterial Osteomyelitis

**DOI:** 10.1002/acr2.11799

**Published:** 2025-01-28

**Authors:** Toni Hospach, Friederike Blankenburg, Anita Heinkele, Kristina Rücklová, Peter Müller‐Abt, Frank Weller‐Heinemann, Laura Buchtala, Jan Maier, Jasmin Kümmerle‐Deschner, Christiane Reiser, Ales Janda, Andreas Urban, Reiner Berendes, Andrea Skrabl‐Baumgartner, Jürgen Brunner, Ralf Trauzeddel, Johannes Peter Haas, Annette Holl‐Wieden, Henner Morbach, Anja Schnabel, Normi Brück, Gabriele Hahn, Wolfgang Emminger, Isabella Valent, Regine Borchers, Christian Klemann, Ilias Tsiflikas, Jens Klotsche, Kirsten Minden, Christian M. Hedrich, Thekla von Kalle

**Affiliations:** ^1^ Olgahospital Klinikum Stuttgart Germany; ^2^ Olgahospital, Klinikum Stuttgart, Germany, and Charles University Prague Czech Republic; ^3^ Prof.‐Hess‐Kinderklinik Bremen Germany; ^4^ Children Medical Practice Leinfelden‐Echterdingen Germany; ^5^ University of Tübingen Tübingen Germany; ^6^ University of Tübingen, Tübingen, Germany, and Landeskrankenhaus Bregenz Bregenz Austria; ^7^ University Medical Center, Ulm University Ulm Germany; ^8^ Klinikum St Marien Amberg Germany; ^9^ Center for Pediatric Rheumatology Landshut Germany; ^10^ Medical University Graz Graz Austria; ^11^ Medical University of Innsbruck, Innsbruck, Austria, and Danube Private University Krems Austria; ^12^ Helios Klinikum Berlin‐Buch Berlin Germany; ^13^ German Center for Pediatric and Adolescent Rheumatology Garmisch‐Partenkirchen Germany; ^14^ University of Wuerzburg Wuerzburg Germany; ^15^ Technische Universität Dresden Germany; ^16^ University of Vienna Vienna Austria; ^17^ University of Augsburg Augsburg Germany; ^18^ University of Leipzig Leipzig Germany; ^19^ Charité Universitätsmedizin Berlin Berlin Germany; ^20^ Charité Universitätsmedizin Berlin and Rheuma‐Forschungszentrum Berlin Berlin Germany; ^21^ University of Liverpool and Alder Hey Children's NHS Foundation Trust Liverpool United Kingdom

## Abstract

**Objective:**

Pulmonary involvement in chronic nonbacterial osteomyelitis (CNO) is rare. Limited awareness results in diagnostic challenges, especially because malignancy or infection needs to be considered.

**Methods:**

Based on a survey shared among centers participating in the *Kerndokumentation Deutsches Rheumaforschungszentrum* (Germany), this study investigated clinical and imaging presentations, demographic features, treatment response and outcomes of pulmonary involvement in CNO (pCNO). Magnetic resonance imaging and computed tomography images were read centrally by an experienced pediatric radiologist.

**Results:**

Twenty‐two patients with pCNO were included in this study. Among patients with CNO, pulmonary involvement was more common in girls (91% vs 62.8%, *P* = 0.006) and patients with multifocal bone lesions (95% vs 65%, *P* <0.001) but was not associated with systemic inflammation or additional organ involvement. Forty‐two pulmonary lesions were counted with a median of two per patient (two to six). They displayed a median size of 1.8 cm (0.3–4.0 cm) and followed mono‐ (40%) and oligo‐focal (60%) patterns representing consolidations or nodules, abutting the pleura in 50%. Although prominent hilar lymph nodes were present (in 19% of patients), no pathologic enlargement (>1 cm) was seen. When available (3 of 22 patients), histology revealed granulomatous inflammation with lymphocyte infiltration. Development and courses of pCNO did not associate with treatments chosen. Complete remission was reported in 60% of patients, partial remission in 20%.

**Conclusion:**

pCNO is usually asymptomatic. Although more common in girls and patients with multifocal CNO, pCNO is not associated with systemic parameters of inflammation or specific organ involvement. Prognosis of pCNO is favorable, and most lesions resolve over time. Thus, a careful watch‐and‐wait strategy may be appropriate.

## INTRODUCTION

Chronic nonbacterial osteomyelitis (CNO) is a noninfectious autoimmune/inflammatory disease that primarily affects bones.^1^, ^2^ Although all age groups can develop CNO, it most commonly affects children and adolescents with a peak onset between 7 and 12 years. CNO covers a clinical spectrum with sometimes singular self‐limiting bone lesions at one end and chronically active or recurrent multifocal lesions at the other end, which are then also referred to as chronic recurrent multifocal osteomyelitis.^1^, ^3^ However, in addition to bones, further organs may be affected. Among extraosseous manifestations, skin (pustulosis, psoriasis, acne, pyoderma, etc), gut (chronic bowel inflammation), and joint involvement have been most frequently reported.^2^, ^4^, ^5^, ^6^, ^7^, ^8^

**Figure 1:**
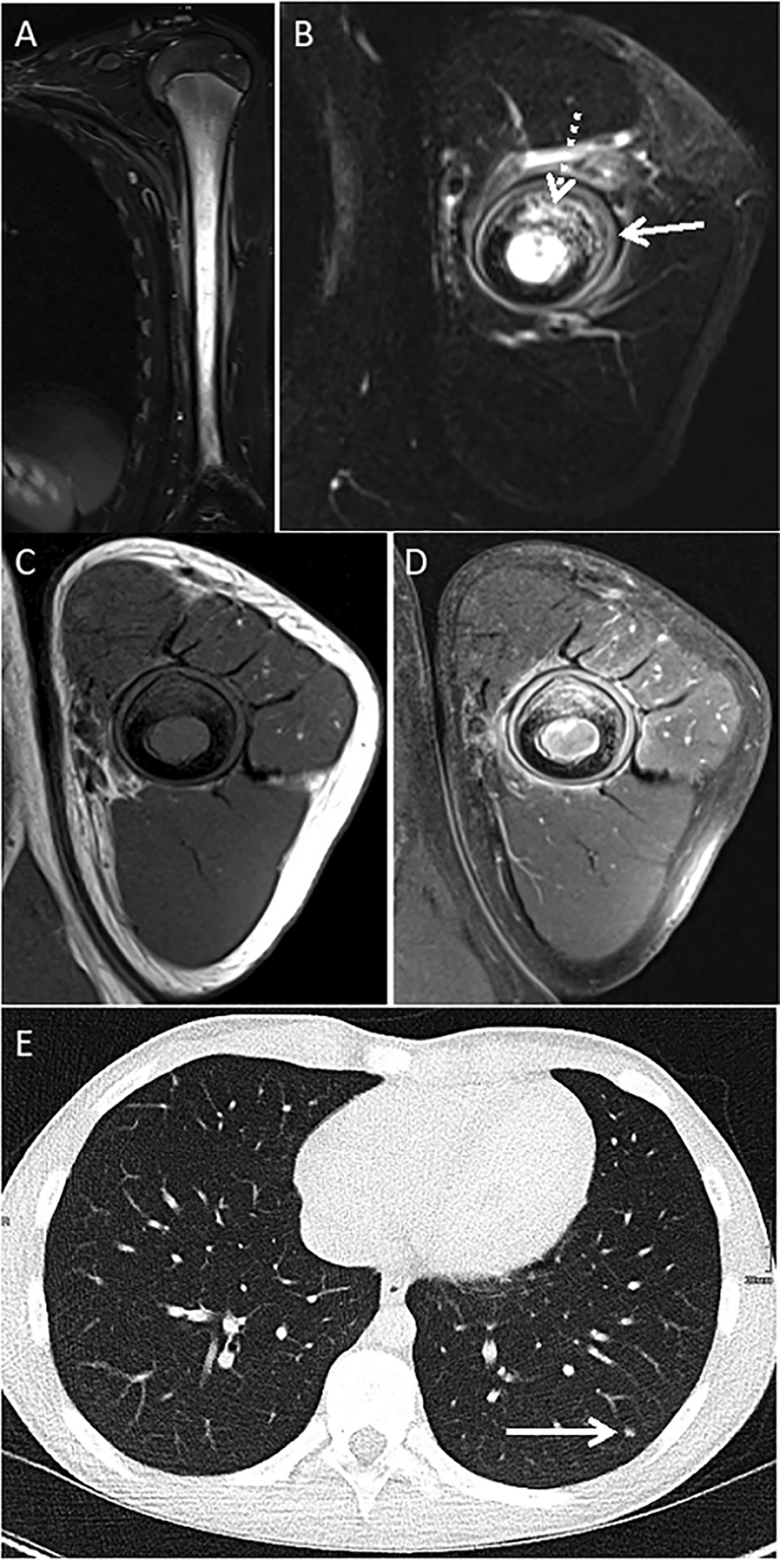
A 14‐year‐old, previously healthy boy developed intermittent pain of his left humerus. An x‐ray was taken six months after the onset of symptoms and revealed an osteolytic lesion. A subsequent MRI showed signs of bone inflammation, osteolysis, and circular bone formation (A–D). By the local pathology service, bone biopsies were interpreted as suggestive of high‐grade osteoblastic osteosarcoma and chest CT revealed the presence of pulmonary infiltrates. Six weeks after starting high‐dose polychemotherapy with methotrexate, doxorubicin, and cisplatin, following EURAMOS/COSS protocols (https://www.sciencedirect.com/science/article/pii/S095980491831534X), national reference pathology results raised doubts in relation to suspected malignancy. A third opinion from an international bone tumor reference center suggested repeating open biopsy, which was suggestive of CNO (chronic inflammatory infiltrates and bone sclerosis) in the absence of malignancy. As a result, the patient was referred to the local pediatric rheumatology service and successfully treated for CNO. (A–D) MRI of the left humerus is shown. (A) Coronal and (B) axial TIRM sequences show hyperintense lesions with cortical destruction (ventral, dotted arrow) with diffuse signal alterations affecting the bone marrow cavity. Circular periosteal bone formation (solid arrow) and limited perifocal soft tissue edema are shown. (C and D) T1 weighted sequences (axial) with (D) contrast enhancement in the bone marrow cavity, the destructed cortical bone, the periosteal new bone, and the perifocal soft tissue are shown. (E) Chest CT with three nodular (only one shown, arrow) opacities in the left lower pulmonary lobe (~5 mm in diameter) is shown. CNO, chronic nonbacterial osteomyelitis; CT, computed tomography; MRI, magnetic resonance imaging; TIRM, turbo inversion recovery magnitude.

In the published literature, pulmonary involvement in CNO (pCNO) has been reported in seven children/adolescents^9^, ^10^, ^11^, ^12^ and two adults.^13^, ^14^ Because of its rarity, little is known about demographic, clinical, and imaging characteristics of pCNO and its clinical course and outcomes. Thus, limited awareness and understanding of pCNO may result in an incorrect diagnosis and aggressive antiproliferative treatments (Figure 1). This manuscript describes demographic, clinical, and morphologic features of pCNO, response to treatment, and outcomes in a national cohort of 22 patients.

## PATIENTS AND METHODS

Patients with pCNO were identified through members of the German Society of Pediatric Rheumatology (GKJR), which also includes Austrian participants. Children and adolescents with CNO, diagnosed by a pediatric rheumatology center, and pulmonary lesions imaging were included into this study. Notably, pulmonary lesions were incidental findings on whole‐body magnetic resonance imaging (WB‐MRI) in all patients reported. The study was approved by the ethics committee, Baden‐Württemberg board of physicians, Stuttgart, Germany. Fully anonymized data will be shared upon reasonable request.

Based on a survey shared among members of the GKJR, clinical and demographic data from patients with pCNO were retrieved from hospital records. The following data were collected: age, sex, age at onset of CNO, age at onset/diagnosis of pulmonary lesions, mode of imaging, respiratory symptoms, (other) extraosseous involvement, (when available) histology results, systemic parameters of inflammation (C‐reactive protein [CRP], erythrocyte sedimentation rate), treatment and response (anti‐infective and/or immunomodulating), and pulmonary lesions at last follow‐up. When necessary (due to incomplete or inconclusive data), corresponding physicians were contacted (by telephone) to confirm and/or complete data (TH). Clinicodemographic data of the patients with pCNO cohort (N = 22) were compared with patients with CNO enrolled in the *Kerndokumentation Deutsches Rheumaforschungszentrum* (Germany) cohort (N = 774).^15^ For statistical analysis, Fisher exact test and Wilcoxon signed‐rank test were performed.

Pulmonary imaging (anonymized), including MRI (all patients) and CT scans (n = 9 of 22 patients), were submitted to the study center (TH, Stuttgart) and reanalyzed by an expert pediatric radiologist with 20 years of experience with CT and MRI of CNO and pulmonary pathology (TvK). Imaging morphology was described following the nomenclature suggested by the *Fleischner* Society^16^, ^17^ as “consolidation” or “nodules.” Additionally, location, size, and number of lesions, as well as abutment to the pleura and/or pleural thickening, was recorded. Hilar lymph nodes were described as “prominent” (larger than contralateral nodes but <1 cm) or “enlarged” (>1 cm).^18^

Our work is informed by patients and their families at all stages. We were tasked with further investigating clinical phenotypes, disease subgroups, and treatment responses through a priority setting event, the International Meeting of CNO and Autoinflammatory Bone Disease, Liverpool (2022), by patients, families, clinicians, and academics involved in CNO research and care.^19^ Results will be fed back to the lay community through our internet presentation and future meetings involving patients and families.

## RESULTS

### Patient demographics

Twenty‐six patients with CNO with pulmonary lesions were identified, all diagnosed between November 1998 and March 2023. Four patients were excluded for the following reasons: In two patients, small atelectases were diagnosed that may have been unrelated to CNO; one patient experienced a respiratory infection that completely resolved after two weeks; one patient was excluded for insufficient data availability. Demographic information and clinical characteristics of the remaining 22 patients with pCNO (Table 1) were compared with a national cohort of patients with CNO (N = 774).^15^ The proportion of female patients was higher among people with pCNO when compared with those with CNO without pulmonary involvement (91% vs 62.8%, *P* = 0.006). Furthermore, at diagnosis, patients with pCNO were slightly older when compared with the national CNO cohort (median 11.5 vs 11 years, *P* <0.001). The proportion of patients with multifocal inflammatory bone lesions was 95% (21 of 22 patients), including 5 patients (23%) with vertebral involvement. Skin manifestations were diagnosed in five patients (23%), all experiencing psoriasis. Gut involvement was seen in one patient (4.5%), who was diagnosed with inflammatory bowel disease (IBD) five months after CNO was diagnosed. No differences were seen in the proportions of patients with skin manifestations (23% vs 14.8%, *P* = 0.36), gut involvement (4.5% vs 1.6%, *P* = 0.3) or arthritis (14 vs 24.5%, *P* = 0.31). Notably, although not reaching statistical significance level, fewer patients with pCNO experienced fevers when compared with the remaining cohort (0 vs 13%, *P* = 0.1).

**Table 1:** Demographics and clinical characteristics of 22 patients with pCNO*

Descriptor	Patients with pCNO included in this study	All patients with CNO included in German National Pediatric Rheumatologic Database^15^	Significance level (*P*)
Sex, female, n/N (%)	20/22 (91)	486/774 (62.8)	0.006^a^
Age at diagnosis in y, median (range)	11.5 (5–16)	11 (1–18)	<0.001^b^
Observation period, mean duration of follow‐up in y	1998–2023, 6.5 (range 1.5–24)	2009–2018, 1	–
Interval between CNO diagnosis and lung involvement in mo, median (range)	8 (0–108)	–	–
Synchronous diagnosis of bone and lung lesions, n/N (%)	9/22 (41)	–	–
Symptomatic respiratory involvement, n	0	–	–
Systemic symptoms (fever/weight loss/night sweats/fatigue), n (%)	0	100 (13), all with fever	0.1^a^
CRP in mg/dL at diagnosis of respiratory involvement, median (range)	<0.5 (<0.5–5.3); >1 mg/dL in 8/22 patients (36.4%)	>1 mg/dL in 107/593 patients (18.0%)^c^	0.03^b^ ^,^ ^c^
Proportion of patients with multifocal CNO (MRI defined), %	95	65	<0.001^b^
Additional organ involvement, %			
Skin involvement	23	14.8	0.36^a^
Gut involvement	4	1.6	0.3^a^
Arthritis	14	24.5	0.31^a^

* CNO, chronic nonbacterial osteomyelitis; CRP, C‐reactive protein; MRI, magnetic resonance imaging; NS, not significant; pCNO, pulmonary involvement in CNO.

^a^ Fisher exact test.

^b^ Wilcoxon signed‐rank test.

^c^ Only limited information was collected in the German National Pediatric Rheumatologic Database (namely, CRP >1 mg/dl vs <1 mg/dL).

The cohort of 22 patients with pCNO in this survey comprised 2.8% of 774 patients with CNO enrolled in the German national registry (*Kerndokumentation Deutsches Rheumaforschungszentrum*).^15^ When considering only patients enrolled to the registry by the centers responding here, 7.6% (22/289) of patients with CNO had pulmonary involvement.

### Diagnostic tests

Several diagnostic tests were applied to exclude differential diagnoses, mainly focusing on infections. To exclude tuberculosis, interferon‐gamma‐release assays were reported in 12 of 22 patients with CNO (55%) and skin tests in 10 of 22 (45%), all of which remained negative. Bronchoalveolar lavages were performed in three patients with CNO, which did not deliver bacterial growth or positive Ziehl‐Neelsen staining.

Three patients underwent lung biopsies, which remained sterile in culture and showed negative results on polymerase chain reaction testing for tuberculosis. All biopsies available (N = 3) displayed granulomatous inflammation with lymphocyte infiltrates. Furthermore, bone biopsies were performed in 10 patients with CNO with lung involvement and revealed mixed cellular infiltrates commonly seen in CNO with granulocytes, monocytes/macrophages, and lymphocytes/plasma cells in the absence of granulomata. Histologic findings did not differ between patients with pCNO and those with CNO in the absence of lung involvement.

**Figure 2:**
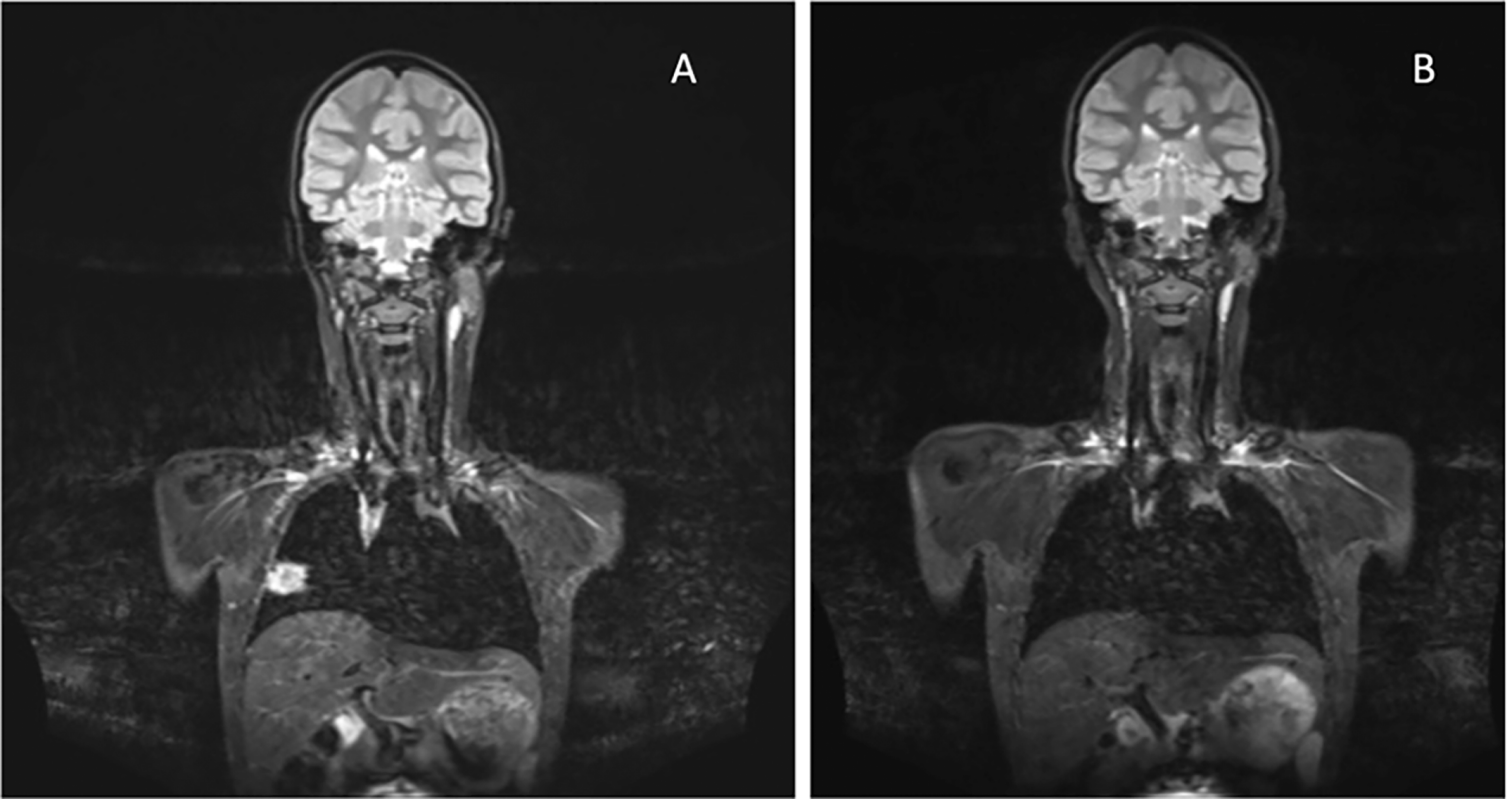
Pulmonary CNO lesions in an 8‐year‐old girl. (A) Coronal STIR MRI sequences reveal a pulmonary lesion in the right middle lobe measuring 2.8 × 2.3 cm, abutting the pleura. (B) Follow‐up MRI in the same patient, 11 months later, shows complete resolution of the pulmonary lesion under treatment with ibuprofen. CNO, chronic nonbacterial osteomyelitis; MRI, magnetic resonance imaging.

Pulmonary involvement was identified, in all cases, based on WB‐MRI aimed primarily at capturing osseous lesions with pulmonary lesions as an incidental finding (Figure 2, Table 2). When available (9 of 22 patients; 40.9%), CT scans were compared with MRI, revealing no differences in morphology, number, or distribution of pulmonary lesions.

**Table 2:** Imaging characteristics of 42 pulmonary lesions in 22 patients*

Descriptor	Results
Imaging technique, n/N (%)	
Whole‐body MRI	22/22 (100)
Consolidation, n/N (%)	17/22 (77.3)
Nodules, n/N (%)	6/22 (27.3)
Abutting pleura, n/N (%)	12/22 (54.5)
Pleural thickening, n/N (%)	7/22 (31.8)
Prominent hilar lymph nodes, n/N (%)	3/16 (18.8)
Monofocal lesions, n/N (%)	9/22 (40.9)
Oligofocal lesions, n/N (%)	13/22 (59.1)
Total number of oligofocal lesions	33
Median number of oligofocal lesions (range)	2 (2–6)
Location of pulmonary lesions, n/N (%)	
Upper lobe	8/42 (19)
Middle lobe	3/42 (7.1)
Lower lobe	31/42 (73.8)
Diameter of lesions in cm, median (range)	1.8 (0.3–4.0)

* MRI, magnetic resonance imaging.

Imaging characteristics of 42 pulmonary lesions in 22 patients (Table 2) included consolidations (77%) and nodules (27%). Pleural involvement was characterized by abutting (55%) and pleural thickening (32%). Prominent hilar lymph nodes (<1 cm) were diagnosed in 19% of patients, but no enlargement >1 cm was seen. Altogether, 42 lesions were counted. In 9 patients (41%), lesions were monofocal; in the remaining 13 patients (59%), 33 lesions were counted with a median number of 2 lesions per patient (range 2–6). Most lesions (74%) were in the lower lobes. The median diameter of the lesion was <2 cm (range 0.3–4.0).

### Treatment

In 9 of 22 patients with pCNO (41%) in whom CNO and pulmonary involvement were diagnosed at the same time, no prior anti‐infective or anti‐inflammatory treatments beyond nonsteroidal anti‐inflammatory drugs (NSAIDs) (n = 8) were recorded. The remaining 13 patients (59%), before the identification of pulmonary lesions, had received, in addition to NSAIDs (13 of 13, 100%), bisphosphonates (5 of 13, 39%) or immune‐modulating drugs, including tumor necrosis factor inhibitors (TNFi) (5 of 13, 39%), methotrexate (4 of 13, 31%), prednisolone (3 of 13, 23%), and/or the IL‐12/23 inhibitor ustekinumab (1 of 13, 8%). When lung disease was diagnosed, treatment with antimicrobial agents was initiated in 5 of 22 patients (23%), none of whom responded (based on short‐term follow‐up with x‐ray imaging or MRI).

### Pulmonary outcomes

Longitudinal data were available in 20 of 22 patients with pCNO (91%); two were lost to follow‐up. Partial response (PR), defined by reduction of lung lesion size but no complete resolution, and complete remission (CR), defined by the absence of residual findings on imaging, was seen in 16 of 20 patients (80%) over a median observational period of 18 months (range 6–96). Partial remission was seen in 4 of 20 patients (20%) with a median observational period of 6 months (range 6–8). Three of four patients had oligofocal pulmonary lesions with a median diameter of 2 cm (range 2–4.5). Treatment for CNO was as follows (before/after diagnosis of pulmonary involvement): pamidronate 0/1, methotrexate 1/0, TNFi 1/1, and NSAIDs 2/1. CR was achieved in 12 of 20 patients (60%) after a median observational period of 6 months (range 2–24). The median duration of documented CR (subject to observational period and/or transition to adult care) was 36 months (range 7–96). Six of twelve patients reaching CR had oligofocal pulmonary lesions with a median diameter of 2 cm (range 2–5). Treatment (before/after) diagnosis of pulmonary involvement was as follows: pamidronate 4/6, prednisolone 3/3, methotrexate 2/2, TNFi 2/1, and NSAIDs 11/5. Two patients exclusively received NSAIDs, and one received no medication at all. A change in treatment as a result of diagnosing pulmonary involvement was reported in 5 of 22 patients (23%) (three received bisphosphonates, one glucocorticoids, and one methotrexate). Treatment modifications (eg, introduction of bisphosphonates), however, were not only due to pulmonary involvement but also to other causes, such as “new” vertebral manifestations.

Of the remaining four patients who did not achieve PR or CR of pulmonary lesions, two experienced a waxing and waning course with phases of remission and recurrence (observational period: 8 and 24 months). One of these patients had six pulmonary lesions with diameters between 0.8 and 2.2 cm. The other two had static oligofocal pulmonary lesions over the observational period (27 and 36 months, median lesion diameter 0.5 cm). Recurrence of pulmonary involvement was seen in three patients, all with lesions affecting areas different to the initial sites. Notably, no patient experienced continuous progression in size or number of lesions over the observational period (median: 19 months, range: 6–96).

## DISCUSSION

Few individual cases of pulmonary involvement in CNO have been reported across the literature,^9^, ^10^, ^11^ but a systematic assessment of its clinical and radiologic presentation, course, and prognosis has been lacking. Because of the severity of differential diagnoses and significant uncertainty, even among expert rheumatologists experienced with the diagnosis and treatment of CNO, a detailed understanding of pCNO and its prognosis and wide awareness among clinicians (including radiologists) are important. Misinterpretation of pCNO as malignancies or infections may lead to uncertainty, parental and patient anxiety, and/or incorrect diagnosis resulting in overtreatment (as described in Figure 1).

This is the first systematic report of a national cohort of patients with pCNO (N = 22), suggesting favorable outcomes. Although overall relatively rare, pCNO may be more common than previously appreciated, affecting up to 7.6% of patients retrieved from the centers participating in the German *Kerndokumentation Deutsches Rheumaforschungszentrum* (2.8% of all patients enrolled in the entire registry). However, the proportion of patients with pCNO may be underestimated because of the retrospective study design. Because targeted thoracic imaging is not generally recommended in CNO, and consequently, the pCNO lesions reported here were identified incidentally on WB‐MRI, additional cases may have been overlooked. On the other hand, the likelihood that inclusion of patients with CNO into the national cohort may be biased toward more severe and (somewhat) untypical cases, potentially favoring overrepresentation of patients with pCNO, also remains possible.

Among patients with pCNO, female predominance (91%) was more pronounced when compared with the entire national cohort (68%, *P* = 0.006) or previous reports from the same region.^6^ Although patients with pulmonary involvement were diagnosed at a slightly younger age when compared to all nationally recorded patients with CNO, differences were marginal (11 vs 11.5 years) and may be explained by the relatively small cohort of patients with pCNO (N = 22). Patients with pCNO more commonly exhibited multifocal bone involvement (95% vs 65%, *P* <0.001), but no differences were seen in relation to additional organ involvement (including skin, gut, and joint involvement) (Table 1). Laboratory parameters of systemic inflammation (CRP) were not markedly altered in the patients with pCNO reported here, suggesting that symptomatic respiratory disease, elevated CRP, etc are not typical of pCNO and therefore likely point toward alternative diagnoses (such as infection or malignancy). Notably, none of the patients with pCNO reported here experienced respiratory symptoms, suggesting that pulmonary involvement in CNO is usually asymptomatic, which agrees with previous individual case reports.^9^, ^10^, ^11^, ^12^ Notably, these observations, to some extent, resemble osseous manifestation, which can also be asymptomatic and even result in vertebral body fractures in previously not symptomatic patients.^6^, ^20^

On imaging studies, pulmonary lesions were characterized by mono‐ (40%) or oligofocal (60%) consolidations or nodules of moderate size (median <2 cm), abutting the pleura in half of patients with pCNO. Most pulmonary lesions (74%) affected lower lobes. In patients who developed new lesions after initial improvement, these appeared at different sites than primary lung manifestations. Prominent hilar lymph nodes (<1 cm) were seen in 19% of patients, none meeting criteria for enlarged lymph nodes (>1 cm). When compared with WB‐MRI, CT scans (available in 9 of 22 patients) showed no differences in morphology, numbers, and distribution of pulmonary lesions. Thus, in most cases, WB‐MRI may be sufficient to diagnose pCNO, reducing radiation exposure of patients.

To exclude differential diagnoses, such as infection or malignancies, histologic work up was performed in 3 of 22 patients. All biopsies available revealed granulomatous inflammation with lymphocyte infiltrates, which was also described in previous case reports.^10^, ^11^, ^13^ Infectious differential diagnoses of granulomatous inflammation, including tuberculosis and/or other mycobacteriosis,^21^ were excluded. Notably, bone lesions in patients with pCNO displayed the CNO typical mixed pattern of innate and adaptive immune infiltrates in the absence of granulomata. The granulomatous character of lung infiltrates was somewhat unexpected because bone inflammation in CNO does typically not involve granulomata.^1^, ^22^, ^23^ However, granulomatous inflammation is a hallmark of Crohn disease, an IBD associated with patients with CNO, and patients with CNO with co‐existing IBD also exhibit nongranulomatous bone inflammation.^1^, ^6^

No associations between pre‐existing or previously accessed treatment and pCNO was observed. Indeed, ~40% of patients exhibited pulmonary lesions at the time of first diagnosis when no medication beyond NSAIDs was recorded. In the remaining 60% of patients, no associations between pCNO onset and accessed treatments were identified. This is of special importance and consideration because of reports describing drug‐induced lung disease after TNFi exposure.^24^, ^25^ A range of treatments were accessed after the identification of pulmonary lesions. The 12 of 20 patients who reached CR were exposed to bisphosphonates, methotrexate, biologic disease‐modifying antirheumatic drugs (including TNFi and ustekinumab), glucocorticoids, and/or NSAIDs before pulmonary lesions were diagnosed. In only five of these patients, treatment was changed after pulmonary lesions were identified, which was usually due to the development of vertebral involvement. Neither size nor number of pulmonary lesions affected disease outcomes. Direct comparisons between patients with pCNO achieving CR and the small group of four patients only reaching PR was complicated by sample size and the median observation period of 6 months (as compared with 36 months in the CR subcohort).

New, previously unreported observations of this study were that pulmonary involvement can occur at any disease stage. Approximately 40% of the patients with CNO reported here exhibited pCNO lesions at the time of diagnosis, whereas the majority developed these later. Furthermore, recurrence of pulmonary lesions has not been reported so far. In three patients of this cohort, new pulmonary lesions appeared after initial resolution, all at different sites. This suggests that aggressive diagnostic procedures or treatment of new pulmonary lesions on MRI may not be necessary. However, reports on reactivation of tuberculosis in patients receiving TNFi treatment should trigger considerations in patients with CNO treated with monoclonal cytokine blocking strategies (and/or other immunomodulating treatments).^26^

Although the study presented here reports the largest available cohort of patients with pCNO, it is limited by a relatively small number of patients (N = 22). Another limitation is the retrospective data acquisition through a survey addressed at experts participating in a national registry. Finally, mainly because of the limited observational period and transition of patients to adult services, long‐term follow‐up data are limited or not available.

**Figure 3:**
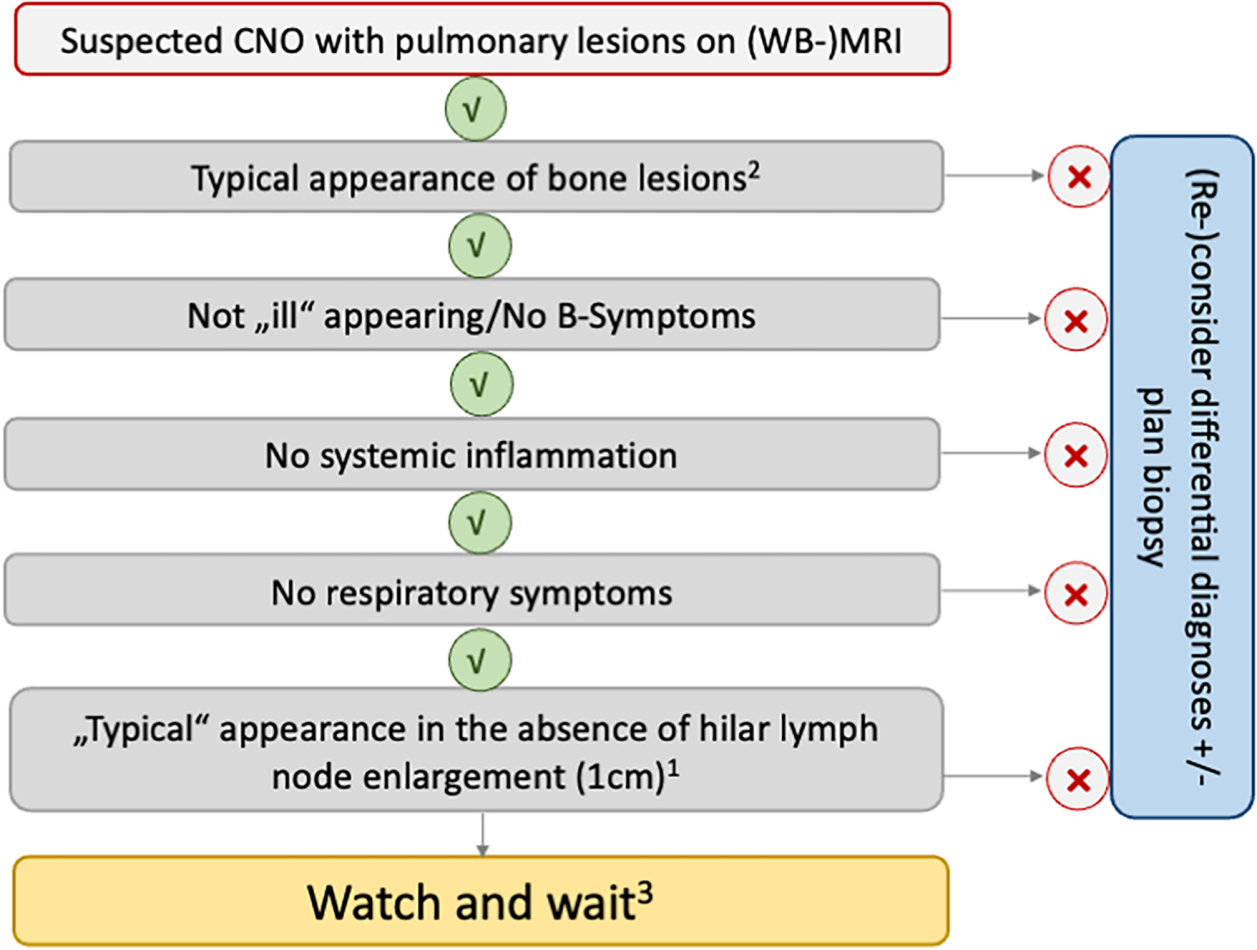
Proposed algorithm for decision‐making in patients with suspected CNO with pulmonary lesions. ^1^Moderately sized (<2 cm) mono‐ or oligofocal lesions, consolidations, nodules ± pleural abutting, hilar lymph nodes not pathologically enlarged (<1 cm). ^2^Polyfocal, symmetric, epi‐/metaphyseal, vertebral, clavicular and/or mandibular involvement, no neurocranial involvement. ^3^Close clinical monitoring (including imaging). CNO, chronic nonbacterial osteomyelitis; WB‐MRI, whole‐body magnetic resonance imaging.

In conclusion, pulmonary involvement in CNO may occur in 3% to 8% of patients and usually remains asymptomatic. Because CNO mimickers (eg, osteosarcoma with metastases) or infections (eg, mycobacterial infections) may manifest with similar pulmonary findings on imaging, they require thorough consideration during the diagnostic process (Figure 3). Pulmonary involvement is associated with female sex and multifocal CNO. Moderately sized mono‐ or oligofocal lesions (<2 cm) with consolidations and/or nodules, the majority located in the lower lobes, with or without pleural abutting are the most common presentations. Hilar lymph nodes may be prominent but are usually not pathologically enlarged (<1 cm). Lesions may be identified on regional MRI or WB‐MRI, and CT imaging may not be necessary in most cases. Independent of treatment, prognosis of pCNO is favorable, and most lesions resolve over time. Thus, in the absence of elevated systemic parameters of inflammation and respiratory symptoms in combination with “typical” findings on imaging (mono‐ or oligofocal lesions <2 cm with consolidations or nodules, with or without pleural abutting, hilar lymph nodes <1 cm), a careful watch‐and‐wait strategy with close monitoring may be appropriate. In the absence of biomarkers for pCNO, CNO and its pulmonary manifestations remain diagnoses of exclusion.

## AUTHOR CONTRIBUTIONS

All authors contributed to at least one of the following manuscript preparation roles: conceptualization AND/OR methodology, software, investigation, formal analysis, data curation, visualization, and validation AND drafting or reviewing/editing the final draft. As corresponding author, Dr Hospach confirms that all authors have provided the final approval of the version to be published, and takes responsibility for the affirmations regarding article submission (eg, not under consideration by another journal), the integrity of the data presented, and the statements regarding compliance with institutional review board/Declaration of Helsinki requirements.

## Supporting information


**Disclosure Form**:
